# A novel assay for drug screening that utilizes the heat shock response of *Caenorhabditis elegans* nematodes

**DOI:** 10.1371/journal.pone.0240255

**Published:** 2020-10-09

**Authors:** Chih-Hsiung Chen, Rahul Patel, Alessandro Bortolami, Federico Sesti

**Affiliations:** 1 Department of Neuroscience and Cell Biology, Rutgers University, Robert Wood Johnson Medical School, Piscataway, NJ, United States of America; 2 Neuroscience Center, University of North Carolina-Chapel Hill, Chapel Hill, NC, United States of America; University of California Riverside, UNITED STATES

## Abstract

Biological organisms respond to environmental stressors by recruiting multiple cellular cascades that act to mitigate damage and ultimately enhance survival. This implies that compounds that interact with any of those pathways might improve organism's survival. Here, we report on an initial attempt to develop a drug screening assay based on the heat shock (HS) response of *Caenorhabditis elegans* nematodes. The protocol works by subjecting the worms to two HS conditions in the absence/presence of the test compounds. Post-heat shock survival is quantified manually or in semi-automatic manner by analyzing z-stack pictures. We blindly screened a cassette of 72 compounds in different developmental stages provided by Eli Lilly through their Open Innovation Drug Discovery program. The analysis indicated that, on average, therapeutically useful drugs increase survival to HS compared to compounds used in non-clinical settings. We developed a formalism that estimates the probability of a compound to enhance survival based on a comparison with a set of parameters calculated from a pool of 35 FDA-approved drugs. The method correctly identified the developmental stages of the Lilly compounds based on their relative abilities to enhance survival to the HS. Taken together these data provide proof of principle that an assay that measures the HS response of *C*. *elegans* can offer physiological and pharmacological insight in a cost- and time-efficient manner.

## Introduction

Drug discovery is the main objective of the pharmaceutical industry. The development of a new drug begins in early stage assays that utilize *in vitro* models such as single-cells. These high-throughput screening assays are cost- and time-efficient but generally unable to recapitulate the complex physiology a compound is likely to encounter in a whole animal [1]. Thus *in vivo* preclinical models are subsequently utilized to assess therapeutic potential and safety of selected candidates, but they are costly and time-consuming. For this reason, many potentially therapeutic molecules do not undergo additional evaluation [2]. Hence, a major challenge is developing intermediate high-throughput assays that maintain cost and time efficiency but provide physiological and pharmacological insight.

To address this problem groups have turned to both established and up-and-coming model organisms and systems including three-dimensional (3D) cell cultures, zebrafish, *Drosophila melanogaster* and *Caenorhabditis elegans*. Mammalian 3D cell culture assays are reported to demonstrate higher fidelity of physiological relevance compared to the traditional two-dimensional cell culture systems [3]. Yet, 3D cell culture systems have encountered obstacles in their incorporation in the contemporary drug discovery process. This includes variability in biologically derived matrices and spheroid heterogeneity that compromise reproducibility [3]. More traditional and well established systems, zebrafish and *Drosophila*, possess attributes that make them suitable models to understand biological mechanisms underlying physiology and disease, including high genetic tractability and advanced organ systems [4, 5]. However, both systems have notable limitations in drug delivery and administration, curbing their potential scalability and affordability [4, 5]. Of the available and well characterized invertebrate model organisms, *C*. *elegans* holds the greatest potential in this space. Drug screening studies using *C*. *elegans* have only recently been undertaken, with the first large-scale screening attempt coming in 2006, but during this time rapid development in screening technology has enabled all liquid handling, full automation and the use of transgenic strains [6–8]. Though, two major hurdles have limited the introduction of the worm as an asset in drug discovery: complicated readouts and animal maintenance [7]. Given the relatively short reproductive cycle of the worm, ~5 days, it becomes difficult to distinguish generations apart as studies progress. Indeed, this limitation is often bypassed by the addition of compounds that inhibit reproduction, at the cost of altering worm physiology and enhancing stress processes [9, 10]. Furthermore, previous approaches have relied on complex behavioral readouts and/or single-protein fluorescence levels to assess compound efficacy, that further limit the automation potential of *C*. *elegans* in drug discovery as they often heavily rely on manually assessment and variable measures, respectively.

To address the boundaries of previous attempts to utilize *C*. *elegans* in drug discovery, we developed an assay based on the heat shock (HS) response [11–13]. The HS response provides an animal model to study a number of conditions including exposure to environmental stresses or pathologic conditions, such as ischemia, inflammation, neurodegeneration, cancer, tissue damage, and infection [14–21]. Thus, by engaging a robust molecular response in which several molecular pathways are recruited to mitigate cellular damage and ultimately enhance survival of the animal, the assay could select potent and effective compounds that interact with these pathways. This argues that compounds that target pathways elicited by the HS may have potential in more complex systems and thus high likelihood of therapeutic and commercial success (druglikeness, [22, 23]). The assay also naturally addresses previous limitations, as it is based on a simple, binary scoring system that evaluates compound efficacy by assessing post-HS survival in a time frame that does not require major animal maintenance.

To determine whether there is a casual relationship between survival to HS and therapeutic potential, we established a collaboration with Eli Lilly through their Open Innovation Drug Discovery program (now discontinued). They assembled a Pathway Exploration Cassette specifically tailored for our assay using previously internally tested and well-documented compounds. The cassette was composed of 72 molecules of diverse therapeutic classes that could potentially target different diseases. Compound identity was unveiled at the end of screening, thus all 72 compounds were blindly screened. Here we report on the results of this analysis and discuss the potential utility of this assay as an intermediary resource between *in vitro* and *in vivo* pharmacology.

## Materials and methods

### Drugs

All drugs and chemicals were purchased from Sigma (St. Louis, MO) or provided by Eli Lilly through their Open Innovation Drug Discovery program.

### Age synchronization

N2 Bristol strain, (source: Sesti) nematodes were age synchronized as described before [24, 25]. Briefly, nematodes were grown in standard 6 cm NGM plates (17 g/liter agar, 3 g/liter NaCl, 2.5 g/liter bactopeptone, 1 mM CaCl_2_, 1 mM MgSO_4_, 5 mg/liter cholesterol, 25 mM potassium phosphate buffer (diluted from a mixture of 132 mM K_2_HPO_4_, 868 mM KH_2_PO_4_, which is expected to have a pH of 6.0) seeded with *Escherichia coli* OP50 (source: Sesti) until a large population of gravid adults was reached. The animals were collected in 1.5 ml Eppendorf tubes, washed in M9 buffer (22 mM KH_2_PO_4_, 22 mM NaH_2_PO_4_, 85 mM NaCl, 1 mM MgSO_4_), and lysed with a solution of freshly mixed 0.25 M NaOH and 1% hypochlorite. The worms were incubated at room temperature for approximately 5 minutes, and then the eggs (and carcasses) were collected by centrifugation at 1500 rpm for 1 min and washed in sterile H_2_O four times. The eggs were incubated overnight in M9 buffer and transferred on 96-well plates containing 150 μl liquid S medium. The use of liquid media allowed us to the following. First, maximize the uptake of compounds, as drugs are dissolved in solution and are ingested along with the food (*C*. *elegans* has a thick cuticle that limits the permeability of compounds). According to Zheng *et al*., drug uptake in liquid media by *C*. *elegans* is comparable to that of mice [26]. Second, provide a more uniform heat distribution during the HS [7]. During incubation at 20°C, worms in liquid S medium were shaken at 350 rpm. One liter of S medium was composed of S Basal medium, 10 mM potassium citrate pH 6, 10 ml trace metals solution, 3 mM CaCl_2_, 3 mM MgSO_4_ with:

- S Basal medium: 5.85 g NaCl, 1 g K_2_ HPO_4_, 6 g KH_2_PO_4_, 1 ml cholesterol (5 mg/ml in ethanol), H_2_O to 1 liter.
- 1 M Potassium citrate pH 6.0: 20 g citric acid monohydrate, 293.5 g tri-potassium citrate monohydrate, H_2_O to 1 liter.
- Trace metals solution: 1.86 g disodium EDTA, 0.69 g FeSO_4_ •7 H_2_O, 0.2 g MnCl2•4 H_2_O, 0.29 g ZnSO_4_ •7 H_2_O, 0.025 g CuSO_4_ •5 H_2_O, H_2_O to 1 liter.

Cultures (500 ml) of *E*. *coli* OP50 bacteria were grown overnight to saturation. Bacteria were harvested by centrifugation (3500 rpm for 10 minutes), washed two times in distilled water and resuspended in S medium (100 mg/ml). The bacterial stock solution was stored at 4°C and diluted in S medium to a final working concentration of 5 mg/ml.

### Heat shock protocol

**Fig 1:**
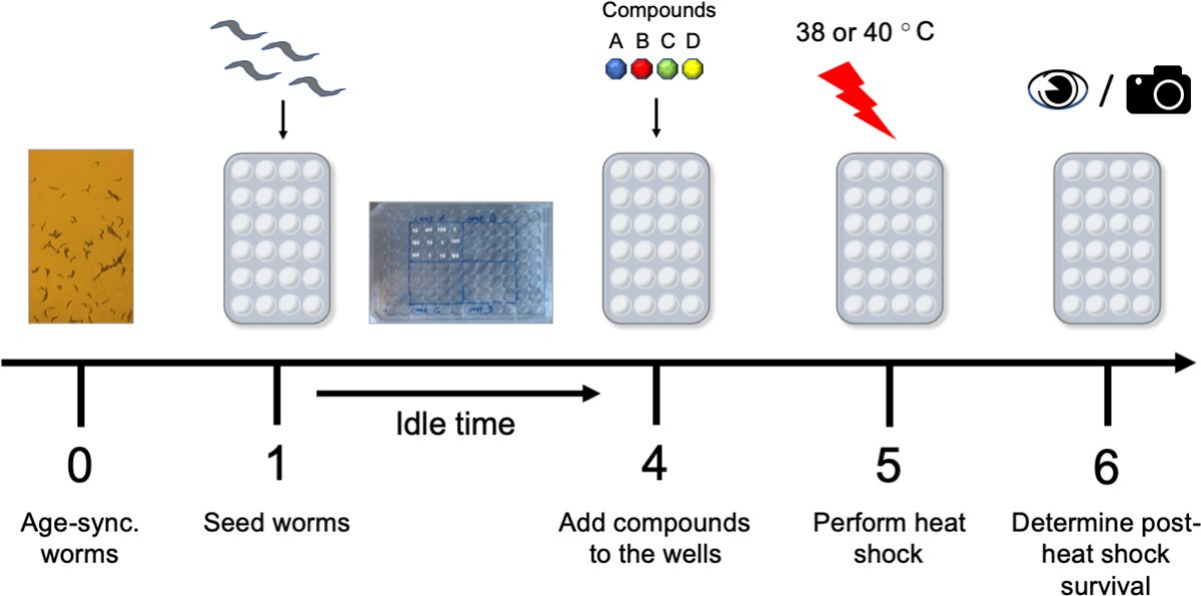
Outline of assay procedure and timeline. A) Day 0—Worms are age-synchronized and newly hatched *larvae* are allowed to hatch overnight. B) Day 1—L0 *larvae* are seeded into individual wells of 96-well plates containing S medium (20–25 worms in 150 μl final volume). C) Day 3—Compounds are added to the wells. Three wells each are supplied with 1, 10 and 100 μM compound (in 1% DMSO) or control (1% DMSO). D) Day 5—Young adult worms are subjected to a heat shock at 38 or 40°C for 3 hours. E) Day 6—Worms are scored for survival (alive worms green color; dead worms grey color) by eye (counting in duplicate) or alternatively semi-automatically, by analyzing z-stack images.

**Fig 2:**
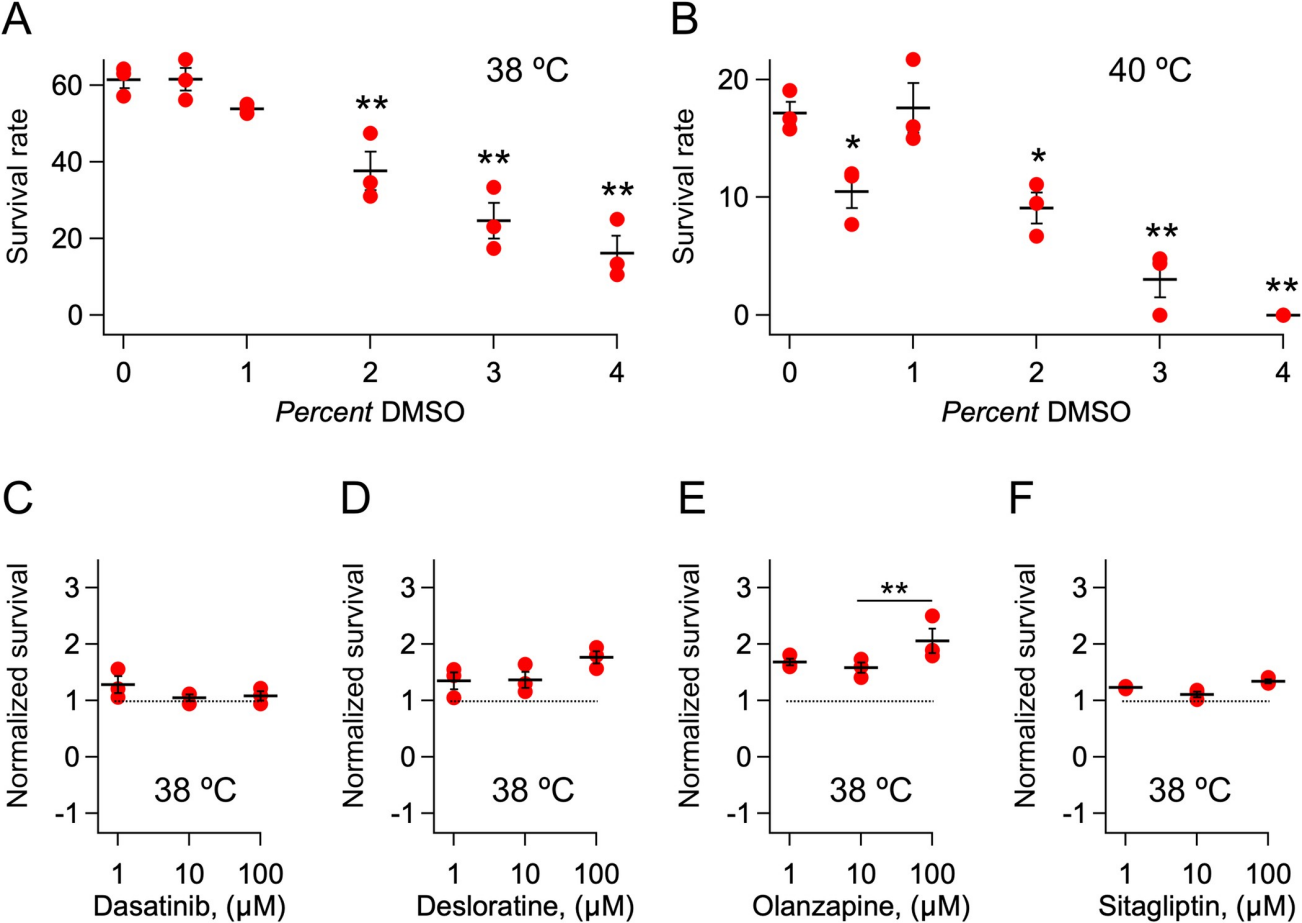
Assay reproducibility. A-B) Survival rate as a function of the concentration of DMSO following a HS at (A) 38°C and (B) 40°C. N = 3 experiments with 3 technical replicate/experiment. C-F) Dose-survival relationships following a HS at 38°C of N = 3 biological experiments (dots) and mean±SEM, for the indicated FDA-approved drugs. Survival data are presented as normalized to control (1% DMSO). *P<0.*05* and **P<0.*01* (one-way ANOVA with Tukey's post-hoc). In (A-B) pairwise comparisons are indicated with respect to control.

The protocol of a typical experiment is described below and is graphically illustrated in Fig 1. To ensure uniform heat distributions and reproducibility the HS was given by placing a 96-well plate containing 5 day-old age synchronized N2 nematodes on a high thermal capacity brick in thermal equilibrium with water [24, 25]. This protocol was developed in part due to previously described challenges of administering reproducible thermal challenges to *C*. *elegans* [27]. Experiments were performed and found similar survival across wells. The duration of the HS, set to 3 hours, was determined empirically as the time necessary to reach steady-state. Longer durations of the HS, (4–8 hrs.) did not significantly decrease survival in agreement with Stegeman *et al*. [28]. Test compounds were dissolved from dimethyl sulfoxide (DMSO) stocks. The concentration of DMSO was maintained uniform at 1% in both the control media and the media containing the compounds. Concentrations of DMSO higher than 1% caused markedly increased mortality compared to control at both 38°C (Fig 2A) and 40°C (Fig 2B). We also noticed that the toxicity of thawed DMSO declined with time, thus care was taken to maintain DMSO freshness by frequently using new DMSO stocks stored at -20°C. In what follows the day indicates both the age of the worms (days 0–3 ~ *larvae*; days 4–6 ~ young adults) and the times of the procedural steps:

- Day 0—Age-synchronize worms.
- Day 1—Aliquots containing ~ 20–25 hatched age-synchronized P0 *larvae* in M9 buffer (typically 10 worms/μl), were pipetted on individual wells of 96-well plates filled with S medium + 5 mg/ml *E*. *coli* OP50 to a 150 μl final volume and incubated at 20°C. Worms were maintained on a shaker throughout experiments to ensure proper aeration and gas exchange.
- Day 4—The test compound was added to nine wells containing the now young adult worms at concentrations 1, 10 or 100 μM in 1% DMSO (3 wells/concentration) in a 150 μl final volume. For each compound, three wells containing 1% DMSO in S medium were used as control (Fig 1 *inset*). Typically, 4 compounds were tested per 96-well plate.
- Day—5 96-well plates containing young adult worms were transferred to a water bath and maintained at 38 or 40°C for 3 hours. At the end of the HS, the worms were returned to the incubator and maintained at 20°C for 24 hours.
- Day 6—Worms were scored for death/survival. The worms were examined under an inverted Olympus IX51 microscope and scored as dead by the absence of movement. Counts were repeated in duplicate. In automation experiments, worms were photographed under an Olympus SZX7 microscope equipped with a digital camera and dedicated Infinity 2 software. Thirty photographs/well were taken in 0.5 sec intervals and automatically z-stacked.

### Mathematical formalism

#### Survival rates

Survival rates, S, in individual wells were calculated as:

$$S(C,T)=\frac{number\;of\;alive\;worms\;in\;the\;well}{total\;number\;of\;worms\;in\;the\;well}$$ (1)

and averaged (3 wells/concentration). T = temperature of the HS (38 or 40°C) and C = concentration of the drug (1, 10 or 100 μM). Data are generally presented as normalized to DMSO control (normalized survival rate) and are indicated as Sn(C,T):

$$Sn(C,T)=\frac{S(C,T)}{S_{DMSO}(0,T)}$$ (2)

where S_DMSO_(0,T) is the survival rate in 1% DMSO (control).

#### Partial derivatives

The partial derivative of the normalized survival rate with respect to the concentration was approximated as:

$$\frac{\partial Sn(C,T)}{\partial C}\approx \langle \frac{\Delta Sn(C,T)}{\Delta C}\rangle =\frac{1}{2}\left(\frac{Sn(10,T)-Sn(1,T)}{0.000009}+\frac{Sn(100,T)-Sn(10,T)}{0.00009}\right)$$ (3)

and is expressed in M^-1^ (mM^-1^ in the graphs). The partial derivative of the normalized survival rate with respect to the temperature of the HS was approximated as:

$$\frac{\partial Sn(C,T)}{\partial T}\approx \frac{\Delta Sn(C,T)}{\Delta T}=\frac{Sn(C,40)-Sn(C,38)}{2}$$ (4)

and is expressed in°C^-1^.

#### φ(C,T) and χ(C,T) functions

The φ(C,T) and *χ*(C,T) functions are binary functions of concentration and HS temperature defined as:

$$\varphi (C,T)=\left\{ \begin{array}{l} 0,\;Sn(C,T)<median\;of\;reference\;Sn(C,T)\\ 1,\;Sn(C,T)\geq median\;of\;reference\;Sn(C,T) \end{array}\right.$$ (5)

and:

$$\chi (C,T)=\left\{ \begin{array}{l} 0,\;\frac{\partial Sn}{\partial x}\leq median\;of\;reference\frac{\partial Sn}{\partial x}\\ 1,\;\frac{\partial Sn}{\partial x}>median\;of\;reference\frac{\partial Sn}{\partial x} \end{array}\right.$$ (6)

where x = C or T.

The probability Γ(φ), expressed in *percent*, that a compound yields φ(C,T) = 0 or φ(C,T) = 1 is:

$$\Gamma (0)=100\left(\frac{compounds\;for\;which\;\varphi =0}{all\;compounds}\right)$$ (7)

$$\Gamma (1)=100\left(\frac{compounds\;for\;which\;\varphi =1}{all\;compounds}\right)$$ (8)

Γ(0)+Γ(1)=100 (9)

and the same applies to Γ(χ). In other words, the Γ function gives the probability that the biological observable of a compound distributes within the lower or upper 50% of the reference drugs.

#### Φ(T), Θ(C) and Ψ functions

In a typical HS experiment a compound was diluted at 3 concentrations (1 μM, 10 μM and 100 μM). There are 8 possible combinations of φ(C,T), for example: 0-0-1; 1-1-0 etc. The Φ(T) function of normalized survival is given by the sum of the combinations of φ(C,T) over the concentrations:

$$\Phi (T)=\sum_{C=1}^{100}\varphi (C,T)=\varphi (1,T)+\varphi (10,T)+\varphi (100,T)$$ (10)

In the above examples Φ(T) = 1 for the combination (0-0-1) and Φ(T) = 2 for the combination (1-1-0). Thus, Φ(T) indicates how many times the normalized survival rate of a test compound is above or below the corresponding reference median and therefore, Φ(T) can assume integer values, n, from 0 to 3. Normalized survival for each concentration was measured at two HS temperatures, 38 and 40°C. Therefore the Θ(C) function of normalized survival is given by the sum of the combinations of φ(C,T) over the temperatures:

$$\Theta (C)=\sum_{T=38}^{40}\varphi (C,T)=\varphi (C,38)+\varphi (C,40)$$ (11)

and Θ(C) can assume integer values, n, from 0 to 2. The same formalism applies to the partial derivatives. There are 3 partial derivatives with respect to the temperature. Therefore a Φ(ΔT) function of the partial derivative with respect to T is defined as:

$$\Phi (\Delta T)=\sum_{C=1}^{100}\chi (C,\Delta T)=\chi (1,\Delta T)+\chi (10,\Delta T)+\chi (100,\Delta T)$$ (12)

and it can assume integer values, n, from 0 to 3. There are 2 partial derivatives with respect to the concentration and a Θ(ΔC) function of the partial derivative with respect to C is defined as:

$$\Theta (\Delta C)=\sum_{T=38}^{40}\chi (\Delta C,T)=\chi (\Delta C,38)+\chi (\Delta C,40)$$ (13)

and it can assume integer values, n, from 0 to 2.

The functions Γ(Φ) and Γ(Θ) express the probabilities, in *percent*, that a compound has a value of Φ = 0,1,2 or 3 and Θ = 0,1 or 2, respectively. Therefore:

$$\sum_{\Phi =0}^{3}\Gamma (\Phi )=100$$ (14)

$$\sum_{\Theta =0}^{2}\Gamma (\Theta )=100$$ (15)

In other words, the function Γ indicates the probability that the normalized survival rates or the partial derivatives of a test compound are n times above or below the corresponding reference medians.

The Ψ function incorporates the φ(C,T) or χ(C,T) values at all concentrations and temperatures to which a compound had been subjected. Therefore the Ψ function of normalized survival is:

$$\Psi =\sum_{C,T}\varphi (C,T)=\sum_{T=38}^{40}\Phi (T)=\sum_{C=1}^{100}\Theta (C)$$ (16)

and it can assume integer values, n, ranging from 0 to 6. Analogously, the Ψ function of the partial derivatives is:

$$\Psi =\sum_{C,T}\chi (C,T)=\Phi (\Delta T)+\Theta (\Delta C)$$ (17)

and it can assume integer values, n, ranging from 0 to 5. The probability for a compound to have a a value of Ψ = 0,1..n, is Γ(Ψ). The probability for a compound to distribute within an interval of Ψ values, Γ(Ψ interval), is given by the sum of the individual Γ(Ψ)s for that interval. For example:

Γ(0−3)=Γ(0)+Γ(1)+Γ(2)+Γ(3) (18)

### Statistical analysis

A single biological experiment, with 3 technical replicates, was carried out per compound. Therefore survival rates are presented as means of the technical replicates and the standard error of the mean (SEM) is not indicated. Experiment-to-experiment variability was tested in triplicate on four FDA-approved drugs, namely, dasatinib, desloratine, olanzapine and sitagliptin (Fig 2C–2F). For all drugs, survival rates varied only moderately in the range of concentrations (1–100 μM) employed. Statistical parameters were calculated using routines freely available on-line. Means, standard errors of the mean (SEMs) and pairwise comparisons between means (Tukey's post-hoc) were calculated using one-way ANalysis Of VAriance (ANOVA) at: https://astatsa.com/OneWay_Anova_with_TukeyHSD/. Medians were calculated at: http://www.alcula.com/calculators/statistics/median/. Statistically significant differences between medians were estimated by the Mood's test available at https://atozmath.com/CONM/NonParaTest.aspx?q=mmt. A two-sample Kolmogorov-Smirnov test (K-S test) was performed to determine whether two samples came from the same distribution (Figs 5 and 6). The K-S test was run at https://www.aatbio.com/tools/kolmogorov-smirnov-k-s-test-calculator.

## Results

### Screening of the Eli Lilly compounds

**Fig 3:**
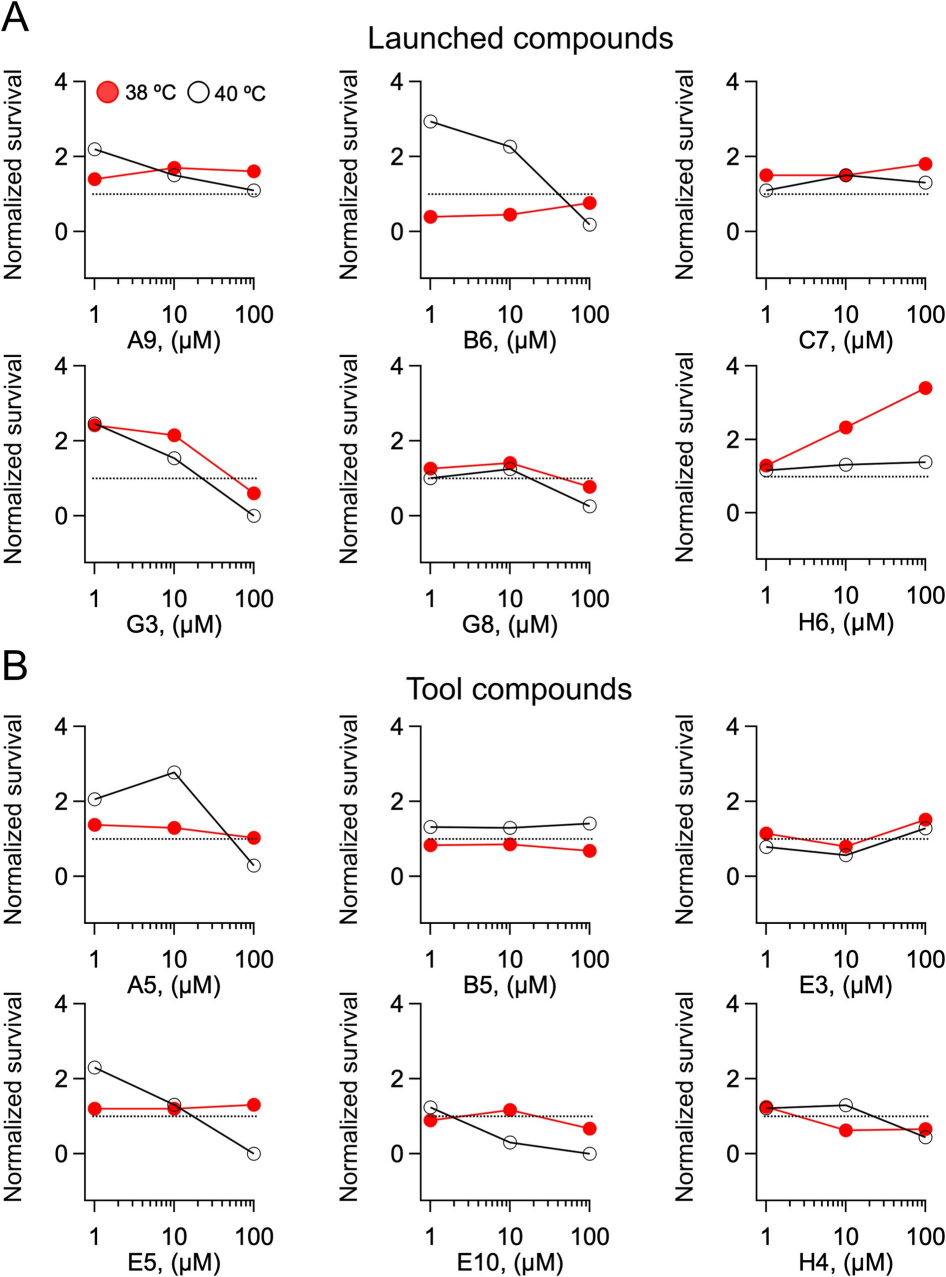
Survival responses to heat shock elicited by launched and tool compounds. A) Dose-normalized survival relationships following a HS at 38°C (red circles) and a HS at 40°C (black hollow circles) for the indicated reference Lilly launched compound. B) Dose-normalized survival relationships following a HS at 38°C (red circles) and a HS at 40°C (black hollow circles) for the indicated reference Lilly tool compound. In all panels, the dotted line indicates normalized survival = 1.

To determine whether there is a relationship between survival to a heat shock (HS) response and therapeutic potential, we screened a pool of compounds provided to us by Eli Lilly through their Open Innovation Drug Discovery program. The Lilly cassette included 17 launched compounds, that is drugs that have received approval from the FDA and are currently in the market and 23 tool compounds, that is compounds that are potent and specific for biological targets used in life science research and non-clinical settings [29] (S1 Table). Without any *a priori* knowledge about the molecules, we blindly assessed how the Lilly compounds affected survival (Eq 1) following heat shocks at 38°C (HS_38_) and at 40°C (HS_40_). Each compound was administered at three concentrations spanning two orders of magnitude (1, 10 and 100 μM) and thus the experimental protocol provided information about six different conditions. Representative examples of normalized survival rates (Eq 2) versus concentration and temperature (dose-normalized survival, DSn relationships) of launched and tool compounds at HS_38_ and HS_40_ are illustrated in Fig 3 and reported in S1 Table. Normalized survival rates broadly varied across compounds, concentrations and temperatures. Overall no major qualitative differences were apparent between the DSn relationships of launched and tool compounds. Therefore, in order to determine whether therapeutic and non-therapeutic compounds affect survival differently in the HS assay, we took a statistical approach.

### Launched compounds increase survival to HS

**Fig 4:**
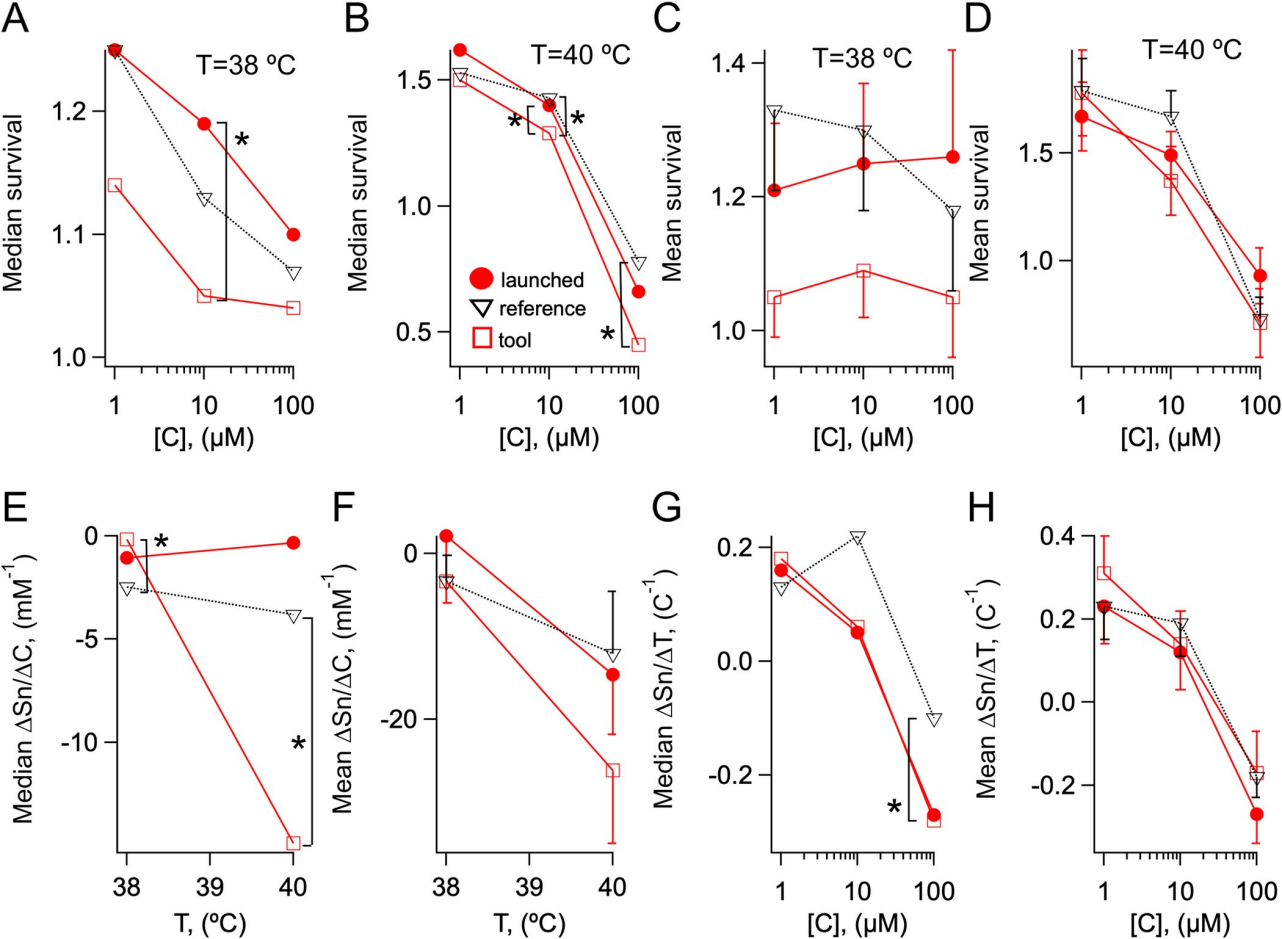
Statistical biological observables of Lilly launched and tool compounds and reference drugs. A-B) Median normalized survivals for launched (red circles), tool (red hollow squares) and reference drugs (black triangles) at (A) HS_38_ and (B) HS_40_ for the indicated concentrations. C-D) Mean normalized survivals for launched (red circles), tool (red hollow squares) and reference drugs (black triangles) at (C) HS_38_ and (D) HS_40_ for the indicated concentrations. E-F) Median (E) and mean (F) partial derivatives of normalized survival with respect to the concentration for launched (red circles), tool (red hollow squares) and reference drugs (black triangles) for the indicated HS temperatures. G-H) Mean (G) and means (H) partial derivatives of normalized survival with respect to the temperature for launched (red circles), tool (red hollow squares) and reference drugs (black triangles) for the indicated concentrations. N = 17, 23 and 35 compounds for respectively, launched and tool compounds and reference drugs. *P<0.*05* (Mood's test).

Fig 4A and 4B illustrate the medians of normalized survival rates of launched and tool compounds. At both HS_38_ and HS_40_, the medians of launched compounds were greater than those of tool compounds. Making a simple assumption that medians randomly have a 50% chance of being either greater or less than one another the probability that this is an adventitious event is P = 2^−6^. Accordingly, the medians of the launched compounds were significantly higher than those of the tool compounds in the medium to high concentration range (10–100 μM) at both HS temperatures (Fig 4A and 4B). We noticed that in the low concentration range (1–10 μM) the medians were greater at HS_40_ than HS_38._ Since the survival rate is normalized to the DMSO control, these values do not mean that raising the temperature increases absolute survival. Rather, they give a measure of the ability of compounds to promote survival when the temperature of the HS is increased. The mean normalized survival rates at HS_38_ and HS_40_ are plotted in Fig 4C and 4D. Overall, launched compounds show a trend to increased survival to a HS compared to tool compounds.

### Therapeutic compounds stabilize survival under variable HS conditions

Since the normalized survival rate varies with the concentration and the temperature, the more robustly beneficial a compound is for worms undergoing HS, the less the normalized survival of the worms should decrease either for increasing temperature at a fixed compound concentration, or for increasing concentration at a fixed temperature and *vice versa*. Therefore the partial derivatives of normalized survival with respect to concentration (Eq 3) and temperature (Eq 4) might differ between therapeutic and non-therapeutic compounds. The medians and means of the partial derivatives with respect to concentration, ∂Sn/∂C, of launched and tool compounds are plotted in Fig 4E and 4F. The derivatives are generally negative and, especially those of tool compounds, strongly temperature dependent. Notably, the derivatives varied less—an indication of enhanced survival—for launched than tool compounds. Mean and median values of the partial derivatives with respect to the temperature, ∂Sn/∂T, are illustrated in Fig 4G and 4H. The derivatives are positive in the low concentration range and become negative at high concentrations, in agreement with the fact that the ∂Sn/∂C are temperature dependent. However, no statistically significant differences were detected between the partial derivatives of normalized survival with respect to the temperature of launched and tool compounds.

Overall, launched and tool compounds enhanced *C*. *elegans*'s survival to a HS. On average, therapeutic compounds seemed to improve survival moderately, but consistently better than non-therapeutic tool compounds.

### FDA-approved drugs recapitulate launched compounds in the HS assay

To determine whether increased survival to HS is a general attribute of therapeutic compounds, we screened a panel of 35 FDA-approved drugs, that we name the reference group, that are employed for the treatment of conditions ranging from metabolic disease to mental disease, to cardiovascular disease to cancer (S2 Table). In the majority of cases, the statistical parameters of the reference drugs, namely median and mean Sn(C,T), ∂Sn/∂C and ∂Sn/∂T, more closely matched those of the Lilly launched compounds than tool compounds (Fig 4).

### Individual biological observables correctly distribute with respect to reference medians

**Fig 5:**
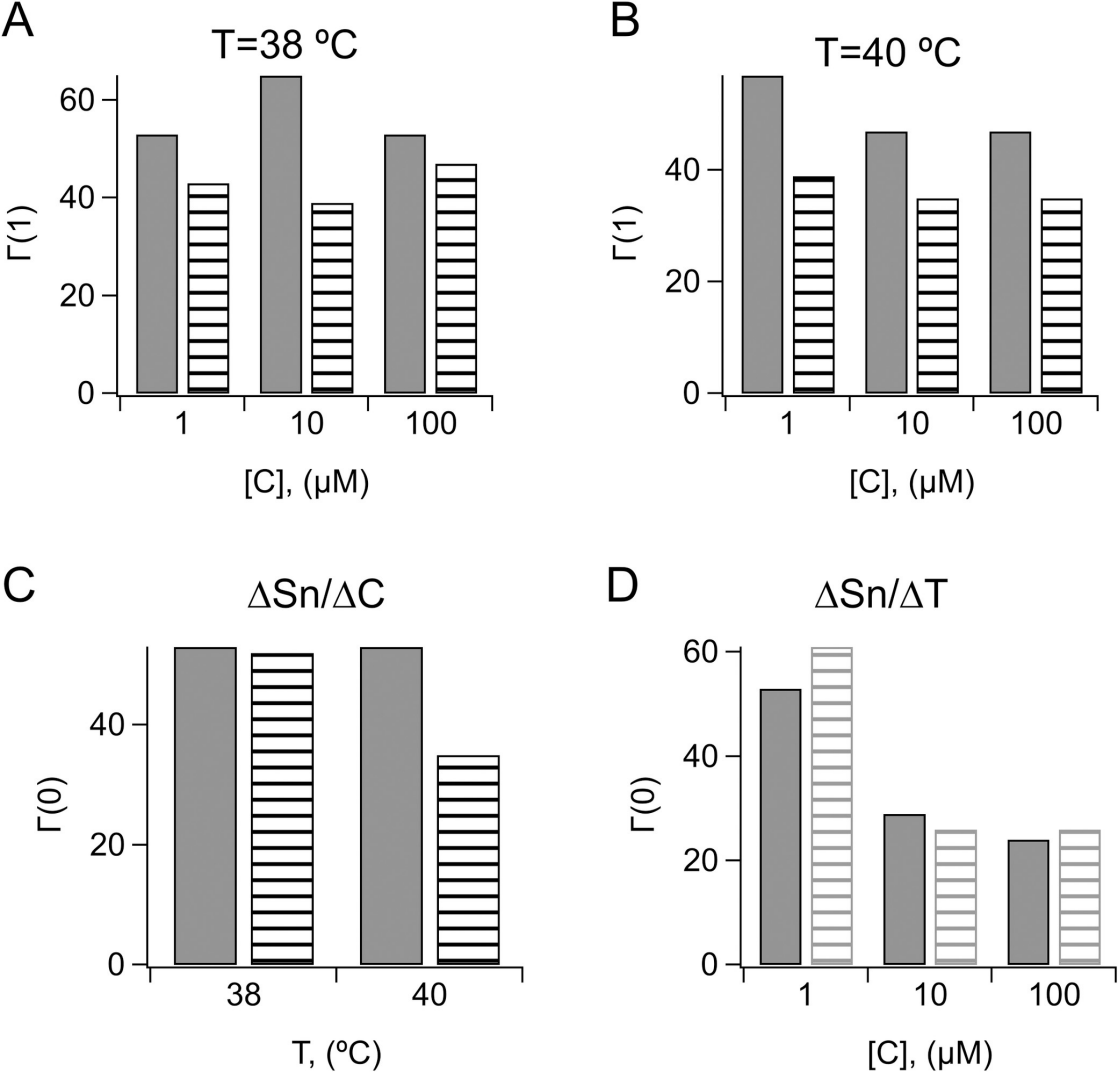
Distributions of launched and tool compounds with respect to the reference medians. A-B) Probabilities for launched (solid) or tool (stripes) compounds to have the value of the normalized survival rate above the corresponding reference median at (A) HS_38_ or (B) HS_40_ for the indicated concentrations. In (A) and (B), *P<0*.*05* (two-sample K-S test). C-D) Probabilities for launched (solid) or tool (stripes) compounds to have the value of the partial derivative of normalized survival with respect to (C) the concentration or (D) the temperature below the corresponding reference medians at the indicated HS temperatures or concentrations.

While, on average, therapeutic drugs exhibited increased survival capacity compared to non-therapeutic compounds, there was large variability at the level of the single compound. This prompted us to seek a probabilistic definition of drug-induced survival to HS. To this end we defined a formalism that gives the probability of a compound to enhance survival based on a comparison with a set of parameters, touchstones, obtained from the reference drugs. We define two binary functions of concentration and temperature, φ(C,T) and χ(C,T), (Eqs 5 and 6) that indicate whether, respectively, the normalized survival rate or a partial derivative of a compound, distribute below or above the median of the reference drugs. Compounds whose normalized survival rates distribute in the upper 50% of normalized survival rates of reference drugs are more likely to enhance survival to HS and therefore the relevant value is φ(C,T) = 1. The probabilities (Eqs 7–9) that a compound scores φ(C,T) = 1, for launched and tool compounds under the experimental range of concentrations and HS temperatures, Γ(1), are shown in Fig 5A and 5B. The Γ(1) values of launched compounds are consistently higher than those of tool compounds, as expected, since the medians of the former are numerically more similar to the reference medians. The same approach was used to calculate the Γ(χ) functions for the partial derivatives. Here, the relevant value was χ(C,T) = 0, as lower variations in normalized survival between changing conditions (of drug concentration or temperature) are indicative of stability under stress and thus increased survival capacity. The Γ(0) values relative to the ∂Sn/∂C are illustrated in Fig 5C and those relative to the ∂Sn/∂T in Fig 5D. In the first case, Γ(0) is greater for launched compounds compared to tool compounds, whereas in the second case the two groups have similar Γ(0) values.

### Survival capacity correlates with the times a compound distributes above or below the reference medians

**Fig 6:**
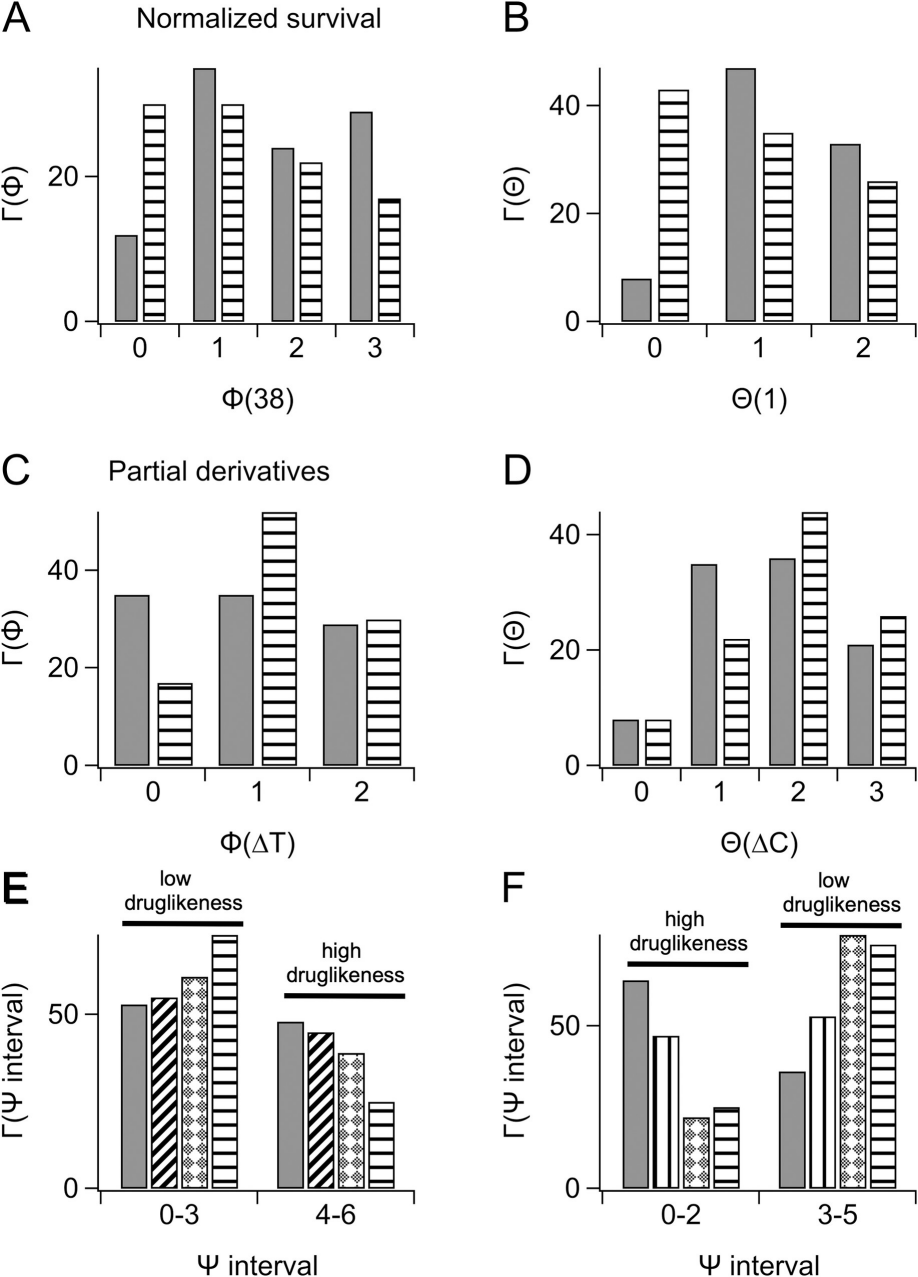
Survival capacity correlates with the times a compound crosses the reference medians. A-B) Probabilities for launched (solid) or tool (stripes) compounds to have the indicated values of: (A) Φ(38), or (B) Θ(1). C-D) Probabilities for launched (solid) or tool (stripes) compounds to have the indicated values of: (C) Φ(ΔT), or (B) Θ(ΔC). E-F) Probabilities for launched (solid), clinical (cross stripes), preclinical (diamonds) or tool (horizontal stripes) compounds to have the indicated value intervals of: (E) Ψ function of normalized survival, or (F) Ψ function of the partial derivatives.

The Γ(φ) and Γ(χ) functions correctly indicated how launched and tool compounds distributed with respect to the reference median for a specific experimental concentration and temperature. We reasoned that the times a compound crosses the reference medians might be indicative of survival capacity. Therefore we defined two new functions of normalized survival, Φ(T) and Θ(C) (Eqs 10 and 11), and two analogous functions of the partial derivatives, Φ(ΔT) and Θ(ΔC) (Eqs 12 and 13), that indicate how many times a test compound is above or below the corresponding reference median over the concentration (1–100 μM) or temperature (38–40°C) ranges employed in our HS experiments. Hence, a compound scoring Φ(38) = 0 in a characteristic HS_38_ experiment, would indicate lower survival capacity relative to a compound scoring Φ(38) = 1, etc.. The probabilities of a test compound to have any Φ(T) or Θ(C) value (and therefore yield normalized survival rates that are above or below the corresponding reference medians n times) are indicated with Γ(Φ) and Γ(Θ) (Eqs 14 and 15). Fig 6A illustrates Γ(Φ) probabilities for normalized survival of launched and tool compounds subjected to a HS_38_ experiment and Fig 6B illustrates the Γ(Θ) probabilities for two HS experiments at 38 and 40°C at one fixed concentration ([C] = 1μM). In all conditions, the values of Γ(0), which indicates the probability to have the lowest survival capacity, are greater for tool compounds than launched compounds, whereas at the other end of the spectrum, Γ(2) and Γ(3) values are greater for launched than tool compounds. Similar distributions were obtained for Φ(40), Θ(10) and Θ(100) (S1 Fig). The analysis of the partial derivatives is illustrated in Fig 6C and 6D. In this case low values of Φ(ΔT) and Θ(ΔC) (Eqs 12 and 13) indicate the propensity to promote survival in the HS, and accordingly the Γ(0) values are greater, for both derivatives, for launched than tool compounds and *vice versa* for the Γ(2) and Γ(3) values.

### An attempt to estimate druglikeness

Since the statistical approach that we developed was able to correctly represent survival to a HS, we hypothesized that the method might inform about the likelihood of a test compound to move forward in development. To obtain proof-of-principle of this idea we attempted to predict druglikeness of the Lilly compounds based on the comparison with the reference medians. The Lilly cassette contained compounds that we did not consider in the previous analysis because their status—therapeutic/non-therapeutic—was not defined. These compounds included 29 developmental candidates either undergoing clinical experimentation (3 compounds in phase III; 6 compounds in phase II and 2 compounds in phase I) or in the preclinical stage (18 compounds). One compound, F7 whose development was not reported, and two compounds, H8 and H9, whose development was discontinued, were removed from the analysis. If our approach was correct the clinical and preclinical groups of compounds should distribute in between tool and launched compounds. We used the Ψ function (Eqs 16 and 17) that measures how many times a compound outperforms the reference medians over all experimental concentrations and temperatures, and given the predictive scope of the attempt, we divided Ψ values into a low and a high therapeutic potential intervals. The low interval of normalized survival was set = 0–3 and the high interval = 4–6. Conversely, the low interval of the partial derivatives was set = 3–5 and the high interval = 0–2 (high/low druglikeness in Fig 6E and 6F). The probabilities of the four groups of Lilly compounds to distribute within the low or high intervals, [Γ(Ψ interval, Eq 18] are illustrated in Fig 6E and 6F. Thus, launched compounds distributed consistently higher in the high intervals followed by clinical, preclinical and tool compounds and *vice versa*. Except clinical compounds, the percentage of compounds that simultaneously distributed in the two intervals of high therapeutic potential, exceeded the 50% threshold, (launched 59%; tools 63%; preclinical 61% and clinical 27%). If we make the assumption that the group of clinical compounds contains more therapeutic compounds than the preclinical group, we can conclude that the four groups of compounds distribute according to their degree of druglikeness.

### Automatic measurement of post-heat shock survival

**Fig 7:**
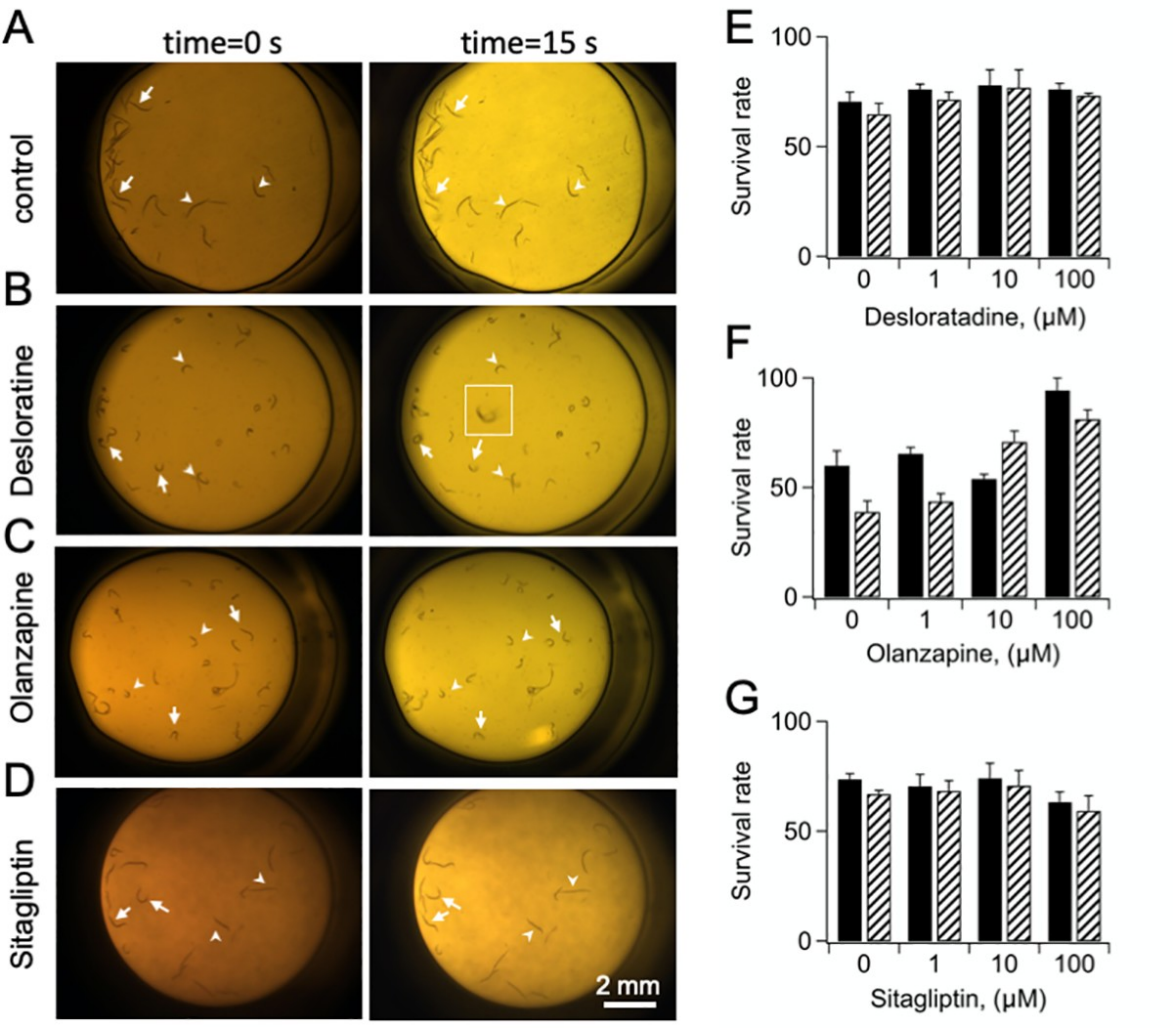
Alive and dead worms can be distinguished in z-stack pictures. A-D) Representative pictures of individual wells containing worms 24 hours post-HS_38_. The pictures on the left were taken at time = 0 and the pictures on the right are z-stacks of 30 pictures taken in 0.5 sec. intervals. For each picture, 2 dead worms (arrows) and two living worms (arrowheads) are indicated. Dead worms maintain sharp images in the z-stack picture, whereas alive, moving worms, appear blurred (panel B *inset*). E-G) Quantification of survival rates measured by eye (solid pattern) or by analyzing z-stack images (stripes) for the indicated reference drugs at HS_38_. Each well was first photographed and then survival rates were measured by eye. Subsequently, survival rates were calculated by analyzing z-stack images in a blind manner. Survival rates were calculated using (Eq 1) and presented as *percent*. N = 2 biological experiments per method with 3 technical replicates/experiment.

One advantage of the HS assay is its binary nature, which can be exploited for automation, a key factor in drug screening. We took advantage of the fact that dead worms are motionless to establish proof-of-principle for a simple, semi-automatic readout method that measures post-heat shock survival on the basis of worms' movements. To this end, we took 30 consecutive pictures in 0.5 sec intervals of single wells 24 hours post-heat shock and z-stacked them (Fig 7A–7D). In the z-stack picture, the images of dead worms are sharp because they did not move; in contrast, the images of alive worms are blurred (*inset* in Fig 7B) or the worms have moved (Fig 7C). We tested this method on 3 reference drugs, desloratadine, olanzopine and sitagliptin that were simultaneously analyzed using eye counting and the z-stack method. The results of these pilot experiments are shown in Fig 7E–7G and show strong correspondence between the survival rates calculated by eye and by the z-stack method.

## Discussion

This study represents an initial attempt to evaluate the potential of therapeutic drugs to enhance the survival of *C*. *elegans* worms to a HS. The rationale is that a HS elicits a robust response in which multiple cellular cascades are recruited to mitigate damage and ultimately enhance survival. Therefore compounds that interact with any of these cascades and as a consequence—improve the survival of the animal—are expected to have high likelihood of therapeutic success. An example is provided by drugs structurally related to tricyclic antidepressants and based on similar pharmacophores. In the human brain tricyclic antidepressants enhance serotonergic transmission by acting primarily as selective serotonin reuptake inhibitors [30]. In *C*. *elegans* these drugs increase lifespan and decrease oxidative stress [31, 32]. All the tricyclic antidepressant tested in this study, namely chlorpromazine, clomipramine, duloxetine and mirtazapine (S2 Table), improved survival above median values. In fact, serotonergic signaling is directly involved in the HS response of the worm [33, 34]. Thus, drugs that are therapeutic in humans can improve the resistance of *C*. *elegans* to a HS by targeting conserved biologic pathways. However, also tool compounds can interact with the same pathways targeted by therapeutic drugs (this was not the case here as launched and tool compounds had distinct targets). This implies that the protective effect of therapeutic drugs is influenced by multiple factors. We put forward the idea that compounds possess an intrinsic "subliminal" toxicity that does not depend by the interaction with a specific pathway and that makes certain molecules incompatible to humans. In this context, the HS acts as a sort of filtering process that selects against subliminal toxicity by subjecting the worms to elevated stress. Initially, we hypothesized that the relationships between the protective effect of a compound and the concentration and/or HS temperature were linear, but soon realized that this is not the case. This led us to develop a probabilistic definition of drug-induced survival to HS in which the *C*. *elegans* assay may be seen as a thermodynamic system that does not inform about what happens at the level of the single molecule or pathway—it only reports changes in macroscopic states such as life and death. Accordingly, we defined macroscopic functions that give the probability of a compound to enhance survival based on a comparison with a set of reference parameters obtained from a pool of therapeutic drugs. Our results indicate that compared to non-therapeutic compounds, therapeutic drugs appear to have a higher tendency to promote survival to a HS. In a preliminary test, the method successfully ranked the Lilly compounds based on their developmental stage. In conclusion, these results support an effort for further develop and validate the HS system and underscore several areas for improvement.

Drug uptake in *C*. *elegans* has traditionally been thought to be relatively inefficient because its cuticle is impermeant to non-water soluble compounds [7]. This was not a concern in this study, as worms were grown in liquid media and all compounds appeared to be absorbed (according to Zheng *et al*., drug uptake in liquid media by *C*. *elegans* is comparable to that of mice [26]). However, we used live OP50 bacteria that could potentially metabolize and degrade a compound thereby lowering its availability to the animal. In future studies the use of dead bacteria as a food source could further maximize drug uptake or in alternative, axenic food could replace the bacteria now that this nutrient has been developed in granular form that the worms can eat more easily [35]. Mutant worms with altered cuticle permeability such as bus-5(*br19*) also represent a valid alternative. Bus-5(*br19*) is particularly suitable for drug discovery studies as it responds well to a variety of toxic chemicals, either soluble and volatile [36]. Given the probabilistic nature of the method, increasing the number of observations would likely strengthen the power of the assay. This could be easily achieved by varying the temperature of the HS and/or increasing the range of the concentrations. However, given the relatively large volumes of compounds (100–200 molecules) that the assay is designed to sample in a hypothetical screening project, the latter objectives will require advanced automation. The method that we have developed, that uses a z-stack picture to identify dead and alive worms turned out to be simple and accurate. Most importantly, this method opens the way to full automation as computer software able to take and read z-stack pictures can be easily implemented.

## Conclusions

In summary, this study shows that the HS of *C*. *elegans* could potentially bridge the gap between *in vitro* and *in vivo* pharmacology without sacrificing the low-cost, and high-throughput qualities of *in vitro* assays. As such this assay not only has the potential to expedite the drug discovery process but can significantly reduce the amount of valuable resources this process traditionally requires.

## Supporting information

S1 Fig Survival capacity for Φ(40), Θ(10) and Θ(100). Probabilities for launched (solid) or tool (stripes) compounds to have the indicated values of: A) Φ(40), B) Θ(10) and C) Θ(100).
(TIF)

S1 Table Dose-normalized survival relationships of the Eli Lilly compounds. Normalized survival rates were calculated according to Eq 2. Abbreviations: L launched; T tool, P preclinical; C-I-III clinical phase I-III; W withdrawn; N/A not available. Acronyms: ALK Anaplastic lymphoma kinase; AP-1 Activator protein 1 transcription factor; BCR-ABL hybrid tyrosine kinase fusion protein; BET Bromodomain and extraterminal domain; BMP Bone morphogenetic protein; CDK Cyclin-dependent kinase; c-Kit tyrosine-protein kinase KIT; c-MET or HGFR Hepatocyte growth factor receptor; DOT1L DOT1-like, histone H3K79 methyltransferase; DYRK1A Dual specificity tyrosine phosphorylation regulated kinase 1A; EGFR Epidermal growth factor receptor; Eph Ephrin receptor; ERR Estrogen-related receptor; FGFR Fibroblast growth factor receptor; FOXM1 Forkhead box protein M1; GABAR γ-aminobutyric acid receptor; GLUT1 Glucose transporter 1; GPER G protein-coupled estrogen receptor; GPR142 G protein-coupled receptor 142; GSI Gluthamine-synthase I; GSK-3β Glycogen synthase kinase 3 beta; HDAC Histone deacetylase; HER or ErbB Erythroblastic oncogene B kinase; JAK3 Janus tyrosine kinase 3; Lck Lymphocyte-specific protein tyrosine kinase; mTOR Mammalian target of rapamycin; NF-κB Nuclear factor kappa-light-chain-enhancer of activated B cells; PDGFR Platelet-derived growth factor receptor; PKC Protein kinase C; PI3K Phosphoinositide 3-kinase; PPARγ Peroxisome proliferator-activated receptor gamma; RAF Proto-oncogene serine/threonine-protein kinase; Src Proto-oncogene tyrosine-protein kinase.
(DOCX)

S2 Table Dose-normalized survival relationships of the reference drugs. Normalized survival rates at HS_38_ (T = 38°C) and at HS_40_ (T = 40°C) for the indicated therapeutic drugs were calculated according to Eq 2.
(DOCX)
