# Supplementary figures and images for: A novel assay for drug screening that utilizes the heat shock response of Caenorhabditis elegans nematodes
Source: PLoS One. 2020 Oct 9;15(10):e0240255. doi: 10.1371/journal.pone.0240255 (PMC7546469; doi:10.1371/journal.pone.0240255)

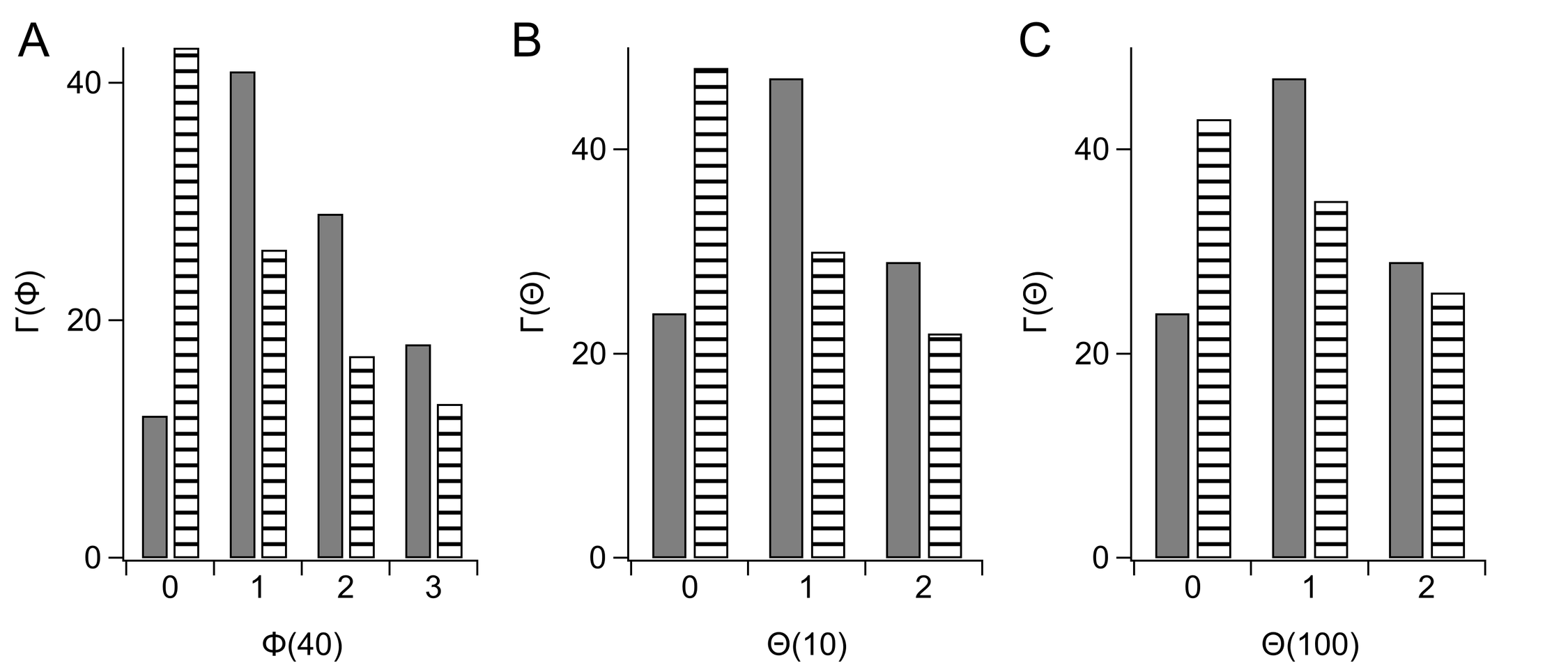

Supplement: S1 Fig — Probabilities for launched (solid) or tool (stripes) compounds to have the indicated values of: A) Φ(40), B) Θ(10) and C) Θ(100). (TIF) [file pone.0240255.s001.tif]
