# Supplementary material for: A novel assay for drug screening that utilizes the heat shock response of Caenorhabditis elegans nematodes
Source: PLoS One. 2020 Oct 9;15(10):e0240255. doi: 10.1371/journal.pone.0240255 (PMC7546469; doi:10.1371/journal.pone.0240255)
Supplement: S1 Table — Normalized survival rates were calculated according to Eq 2. Abbreviations: L launched; T tool, P preclinical; C-I-III clinical phase I-III; W withdrawn; N/A not available. Acronyms: ALK Anaplastic lymphoma kinase; AP-1 Activator protein 1 transcription factor; BCR-ABL hybrid tyrosine kinase fusion protein; BET Bromodomain and extraterminal domain; BMP Bone morphogenetic protein; CDK Cyclin-dependent kinase; c-Kit tyrosine-protein kinase KIT; c-MET or HGFR Hepatocyte growth factor receptor; DOT1L DOT1-like, histone H3K79 methyltransferase; DYRK1A Dual specificity tyrosine phosphorylation regulated kinase 1A; EGFR Epidermal growth factor receptor; Eph Ephrin receptor; ERR Estrogen-related receptor; FGFR Fibroblast growth factor receptor; FOXM1 Forkhead box protein M1; GABAR γ-aminobutyric acid receptor; GLUT1 Glucose transporter 1; GPER G protein-coupled estrogen receptor; GPR142 G protein-coupled receptor 142; GSI Gluthamine-synthase I; GSK-3β Glycogen synthase kinase 3 beta; HDAC Histone deacetylase; HER or ErbB Erythroblastic oncogene B kinase; JAK3 Janus tyrosine kinase 3; Lck Lymphocyte-specific protein tyrosine kinase; mTOR Mammalian target of rapamycin; NF-κB Nuclear factor kappa-light-chain-enhancer of activated B cells; PDGFR Platelet-derived growth factor receptor; PKC Protein kinase C; PI3K Phosphoinositide 3-kinase; PPARγ Peroxisome proliferator-activated receptor gamma; RAF Proto-oncogene serine/threonine-protein kinase; Src Proto-oncogene tyrosine-protein kinase. (DOCX) [file pone.0240255.s002.docx]

|  |  |  |  |  |  |  |  |  |
| --- | --- | --- | --- | --- | --- | --- | --- | --- |
| S1 TABLE Dose-normalized survival relationships of the Eli Lilly compounds | | | | | | | | |
|  | **T= 38 °C** | | | **T= 40 °C** | | |  |  |
| name | **concentration, (μM)** | | | | | | **status** | **target** |
|  | 1.0 | 10.0 | 100.0 | 1.0 | 10.0 | 100.0 |  |  |
| A2 | 1.81 | 1.14 | 1.16 | 2.22 | 0.82 | 0.09 | C-III | CDK1, CDK2, CDK9 |
| A3 | 0.87 | 1.19 | 1.56 | 1.14 | 1.44 | 0.10 | L | c-Met, ALK, HGFR |
| A4 | 0.98 | 1.04 | 0.79 | 1.14 | 1.09 | 1.65 | T | GPR142 |
| A5 | 1.37 | 1.29 | 1.03 | 2.06 | 2.77 | 0.29 | T | Na^+^-glucose cotransporters |
| A6 | 0.92 | 0.81 | 0.58 | 1.27 | 1.01 | 0.69 | T | GSI |
| A7 | 1.51 | 1.21 | 1.00 | 2.97 | 2.22 | 2.98 | C-II | pan cdk |
| A8 | 1.45 | 2.12 | 1.62 | 1.54 | 0.88 | 0.21 | C-II | PI3K, mTOR |
| A9 | 1.40 | 1.70 | 1.60 | 2.20 | 1.50 | 1.10 | L | Beta-adrenergic |
| A10 | 1.25 | 0.84 | 0.81 | 2.43 | 1.40 | 0.00 | L | N/A |
| B2 | 0.93 | 0.89 | 1.10 | 1.70 | 1.40 | 0.51 | L | ALK, EGFR |
| B3 | 1.30 | 1.30 | 1.60 | 1.60 | 1.30 | 1.20 | P | GPR142 |
| B4 | 1.00 | 0.74 | 0.93 | 0.73 | 0.47 | 0.17 | P | N/A |
| B5 | 0.83 | 0.85 | 0.68 | 1.32 | 1.29 | 1.41 | T | CDK1/cyclin B and GSK-3β |
| B6 | 0.39 | 0.45 | 0.76 | 2.93 | 2.26 | 0.18 | L | Bcr-Abl, Src, pan-Eph, Lck, |
| B7 | 0.77 | 0.94 | 1.80 | 0.94 | 1.06 | 1.25 | T | N/A |
| B8 | 0.83 | 0.47 | 0.48 | 3.14 | 3.75 | 3.11 | T | GABARa |
| B9 | 0.91 | 0.91 | 0.85 | 0.36 | 0.75 | 1.42 | C-III | HDAC1 and HDAC3 |
| B10 | 1.04 | 1.32 | 1.45 | 1.40 | 0.82 | 0.81 | P | BMP4 |
| C2 | 1.60 | 1.50 | 0.92 | 3.50 | 1.90 | 0.00 | T | ERRβ and ERRγ |
| C3 | 1.10 | 1.20 | 1.20 | 2.00 | 1.50 | 0.57 | P | Histone deacetylase |
| C4 | 1.50 | 1.40 | 1.20 | 1.50 | 1.30 | 1.20 | C-II | BET bromodomain |
| C5 | 1.11 | 1.03 | 0.98 | 0.65 | 0.62 | 0.57 | T | GPER |
| C6 | 1.01 | 1.50 | 0.94 | 1.03 | 1.36 | 1.05 | L | N/A |
| C7 | 1.50 | 1.50 | 1.80 | 1.10 | 1.50 | 1.30 | L | Class I and II HDAC |
| C8 | 0.83 | 0.76 | 1.17 | 2.30 | 0.32 | 0.07 | P | cAMP and cGMP phosphodiesterases |
| C9 | 1.02 | 0.66 | 0.55 | 1.13 | 0.00 | 0.00 | P | L-type Ca^2+^ channel |
| C10 | 1.39 | 1.43 | 1.32 | 2.09 | 1.55 | 1.83 | T | CK1/TGFbR1 |
| D2 | 1.5 | 1.50 | 1.50 | 1.00 | 1.20 | 0.73 | P | HDAC |
| D3 | 1.00 | 1.10 | 1.10 | 1.50 | 1.00 | 0.11 | T | Adiponectin receptor |
| D4 | 1.13 | 1.46 | 1.03 | 0.23 | 0.66 | 1.33 | P | soluble guanylyl cyclase |
| D5 | 1.21 | 1.20 | 1.21 | 2.33 | 2.39 | 0.00 | P | N/A |
| D6 | 1.48 | 1.28 | 1.77 | 1.62 | 1.65 | 1.31 | L | GPR142 |
| D7 | 1.07 | 0.89 | 1.49 | 1.61 | 1.61 | 1.55 | L | PKA |
| D8 | 0.82 | 0.86 | 0.77 | 1.15 | 0.96 | 0.85 | C-II | SIRT1 histone deacetylase |
| D9 | 1.35 | 1.05 | 1.04 | 1.52 | 1.34 | 0.45 | T | Estrogen receptor |
| D10 | 0.44 | 0.49 | 0.50 | 0.93 | 0.96 | 1.21 | C-III | adenosine A1 receptor |
| E2 | 1.34 | 1.17 | 0.75 | 1.95 | 1.27 | 1.24 | L | c-Kit, FGFR, PDGFR, EGFR |
| E3 | 1.14 | 0.79 | 1.51 | 0.78 | 0.56 | 1.28 | T | FOXM1 |
| E4 | 0.62 | 1.53 | 1.86 | 2.44 | 1.98 | 1.46 | C-II | dual ATP PI 3-K/mTOR |
| E5 | 1.2 | 1.2 | 1.3 | 2.30 | 1.30 | 0.00 | T | Smoothened (Smo) receptor |
| E6 | 0.49 | 0.71 | 0.64 | 1.39 | 1.01 | 1.10 | P | NADPH oxidase |
| E7 | 0.75 | 0.47 | 0.20 | 1.34 | 0.58 | 0.31 | T | phospholipase C |
| E8 | 1.12 | 0.80 | 1.20 | 1.63 | 0.84 | 0.66 | L | polyADP-ribose synthetase |
| E9 | 1.19 | 1.27 | 0.76 | 0.78 | 1.21 | 1.02 | L | N/A |
| E10 | 0.89 | 1.17 | 0.67 | 1.23 | 0.30 | 0.00 | T | JMJD3/UTX histone demethylase |
| F2 | 1.05 | 0.95 | 0.89 | 1.28 | 1.10 | 0.04 | C-I | GPR142 |
| F3 | 1.36 | 2.53 | 3.69 | 0.80 | 1.33 | 1.11 | P | adenosine kinase inhibitor, phosphodiesterase 3 |
| F4 | 1.59 | 1.31 | 0.22 | 1.48 | 1.20 | 0.00 | P | NF-κB and AP-1 |
| F5 | 1.30 | 1.10 | 1.20 | 4.40 | 1.60 | 0.30 | T | N/A |
| F6 | 1.62 | 1.86 | 2.29 | 3.08 | 2.29 | 0.63 | T | GPR142 |
| F7 | 0.96 | 1.16 | 0.82 | 0.87 | 0.83 | 1.27 | N/A* | JAK3, GSK-3β, PKCα and PKCθ |
| F8 | 0.87 | 0.73 | 0.96 | 1.18 | 0.92 | 0.17 | L | B,C-Raf |
| F9 | 1.10 | 1.10 | 1.20 | 0.30 | 1.30 | 1.80 | C-I | DOT1L |
| F10 | 1.80 | 1.60 | 1.20 | 5.14 | 4.69 | 0.00 | P | DAG kinase |
| G2 | 0.86 | 0.98 | 0.80 | 1.84 | 1.31 | 0.12 | T | DYRK1A |
| G3 | 2.41 | 2.15 | 0.60 | 2.46 | 1.54 | 0.00 | L | pan HER |
| G4 | 1.26 | 1.18 | 1.20 | 2.50 | 2.80 | 0.52 | L | EGFR (ErbB1), HER2 (ErbB2), HER4 (ErbB4) |
| G5 | 1.60 | 1.43 | 1.05 | 1.50 | 0.85 | 0.00 | T | GSI |
| G6 | 2.30 | 1.70 | 2.60 | 0.60 | 1.00 | 0.40 | P | DOT1L histone methyltransferase |
| G7 | 1.32 | 1.53 | 1.23 | 1.66 | 1.52 | 1.00 | T | PI 3-kinase β |
| G8 | 1.26 | 1.41 | 0.77 | 1.00 | 1.24 | 0.25 | L | GPR142; BTK, ITK |
| G9 | 1.44 | 1.54 | 1.82 | 1.48 | 1.48 | 0.38 | P | BET bromodomain |
| G10 | 0.52 | 0.66 | 0.55 | 2.30 | 0.32 | 0.07 | P | NF-κB, GPBAR |
| H2 | 1.40 | 1.50 | 1.30 | 1.20 | 1.10 | 0.00 | T | stearoyl-CoA desaturase 1 |
| H3 | 0.92 | 1.28 | 1.28 | 0.91 | 0.97 | 0.39 | P | EZH2 histone methyltransferase |
| H4 | 1.25 | 0.62 | 0.66 | 1.21 | 1.29 | 0.43 | T | GLUT1 |
| H5 | 0.95 | 1.01 | 0.95 | 1.41 | 1.38 | 1.75 | C-II | GPR142 |
| H6 | 1.29 | 2.32 | 3.40 | 1.16 | 1.31 | 1.38 | L | mTOR |
| H7 | 0.96 | 0.97 | 1.13 | 1.16 | 1.44 | 0.87 | T | N/A |
| H8 | 1.59 | 1.46 | 1.24 | 1.63 | 1.26 | 0.28 | W | EGFR |
| H9 | 1.75 | 1.47 | 0.83 | 0.76 | 0.83 | 0.38 | W | PPARγ |
| H10 | 1.39 | 1.29 | 1.50 | 2.11 | 2.00 | 1.79 | P | androgen receptor |
