# Supplementary material for: A novel assay for drug screening that utilizes the heat shock response of Caenorhabditis elegans nematodes
Source: PLoS One. 2020 Oct 9;15(10):e0240255. doi: 10.1371/journal.pone.0240255 (PMC7546469; doi:10.1371/journal.pone.0240255)
Supplement: S2 Table — Normalized survival rates at HS38 (T = 38°C) and at HS40 (T = 40°C) for the indicated therapeutic drugs were calculated according to Eq 2. (DOCX) [file pone.0240255.s003.docx]

| S2 TABLE Dose-normalized survival relationships of the reference drugs | | | | | | | |
| --- | --- | --- | --- | --- | --- | --- | --- |
|  |  | **T= 38 °C** | | | **T= 40 °C** | | |
| drug | **treatment** | **concentration, (μM)** | | | | | |
|  |  | 1 | 10 | 100 | 1 | 10 | 100 |
| Abemaciclib | breast cancer | 1.02 | 1.26 | 1.45 | 1.06 | 1.01 | 0.05 |
| Alpelisib | breast cancer | 0.60 | 0.65 | 0.93 | 1.35 | 1.28 | 0.83 |
| Bariticinib | rheumatoid arthritis | 1.08 | 1.13 | 1.07 | 0.73 | 0.89 | 0.45 |
| Canagliflozin | anti-diabetic (type 2) | 2.01 | 1.08 | 0.12 | 4.90 | 1.68 | 0.00 |
| Chlorpromazole | anti-psychotic | 1.45 | 1.39 | 0.54 | 2.29 | 2.02 | 0.09 |
| Clomipramine | nerve pain/obsessive-compulsive disorder | 2.43 | 2.94 | 1.44 | 1.86 | 1.23 | 0.00 |
| Dasatinib | leukemia | 1.46 | 1.2 | 1.43 | 1.06 | 1.23 | 0.37 |
| Desloratadine | anti-allergic | 1.37 | 1.66 | 1.61 | 1.65 | 1.43 | 1.45 |
| Digoxin | anti-arrhythmic | 0.90 | 0.96 | 1.07 | 1.58 | 1.54 | 1.03 |
| Duloxetine | nerve pain/antidepressant | 1.59 | 1.75 | 0.76 | 1.53 | 0.82 | 0.00 |
| Empagliflozin | anti-diabetic (type 2) | 0.94 | 0.96 | 1.35 | 1.60 | 1.20 | 0.15 |
| Entrectinib | lung cancer | 0.86 | 1.10 | 0.79 | 1.08 | 1.69 | 0.78 |
| Erdafitinib | anti-cancer | 0.79 | 0.79 | 0.88 | 1.54 | 1.33 | 0.91 |
| Finasteride | benign prostatic hyperplasia; hair loss | 1.15 | 0.92 | 0.69 | 1.46 | 1.70 | 1.46 |
| Fluoxetine | anti-depressive | 0.98 | 1.28 | 0.43 | 1.10 | 1.40 | 0.14 |
| Gilteritinib | anti-cancer | 1.55 | 1.27 | 1.08 | 1.68 | 1.56 | 1.16 |
| Glyburide | anti-diabetic (type 2) | 0.77 | 1.08 | 1.62 | 3.29 | 3.73 | 1.05 |
| Ibuprofen | nonsteroidal anti-inflammatory | 1.40 | 1.19 | 0.70 | 3.37 | 2.20 | 0.00 |
| Istradefylline | anti-parkinson (anti-bradykinetics) | 0.54 | 0.80 | 1.12 | 2.96 | 3.60 | 0.16 |
| Lamivudine | antiviral | 1.61 | 0.88 | 1.71 | 1.16 | 1.43 | 0.52 |
| Lisinopril | ACE inhibitor (high blood pressure) | 1.25 | 1.67 | 1.30 | 1.09 | 1.11 | 0.81 |
| Metformin | anti-diabetic (type 2) | 1.73 | 1.61 | 0.03 | 1.5 | 1.43 | 0.94 |
| Mirtazapine | anti depressive | 4.41 | 4.41 | 4.21 | 2.72 | 2.49 | 2.17 |
| Olanzapine | anti psychotic | 1.68 | 2.18 | 2.19 | 1.43 | 2.83 | 2.48 |
| Pitolisant | narcolepsy | 1.18 | 0.96 | 1.10 | 3.33 | 2.54 | 0.79 |
| Pretomanid | tuberculosis antibiotic | 1.44 | 1.21 | 1.02 | 3.01 | 1.24 | 1.36 |
| Prucalopride | chronic constipation | 1.32 | 1.12 | 0.94 | 1.42 | 1.30 | 0.95 |
| Rizatripan | migraine headaches | 1.35 | 0.68 | 0.57 | 1.23 | 1.73 | 0.50 |
| Siponimod | multiple sclerosis | 0.91 | 0.83 | 0.51 | 1.21 | 1.26 | 0.31 |
| Sitagliptin | anti-diabetic | 1.67 | 1.5 | 1.51 | 1.04 | 1.11 | 0.58 |
| Tamsulosin | benign prostatic hyperplasia | 1.75 | 1.44 | 1.17 | 1.45 | 1.58 | 0.98 |
| Tanespimycin | antitumor antibiotic | 0.95 | 0.89 | 0.80 | 1.53 | 1.66 | 0.84 |
| Upadacitinib | rheumatoid arthritis | 1.0 | 1.22 | 1.25 | 1.55 | 2.34 | 1.67 |
